# SemNet: Learning semantic attributes for human activity recognition with deep belief networks

**DOI:** 10.3389/fdata.2022.879389

**Published:** 2022-08-30

**Authors:** Shanmuga Venkatachalam, Harideep Nair, Ming Zeng, Cathy Shunwen Tan, Ole J. Mengshoel, John Paul Shen

**Affiliations:** ^1^Department of ECE, Carnegie Mellon University, Pittsburgh, PA, United States; ^2^Department of ECE, Anderson School of Management, University of California, Los Angeles, Los Angeles, CA, United States; ^3^Department of Computer Science, Norwegian University of Science and Technology (NTNU), Trondheim, Norway

**Keywords:** human activity recognition, deep belief networks, semantic mid-level features, ubiquitous computing, multimodal sensing, artificial intelligence, internet of things

## Abstract

Human Activity Recognition (HAR) is a prominent application in mobile computing and Internet of Things (IoT) that aims to detect human activities based on multimodal sensor signals generated as a result of diverse body movements. Human physical activities are typically composed of simple actions (such as “arm up”, “arm down”, “arm curl”, etc.), referred to as *semantic* features. Such abstract semantic features, in contrast to high-level activities (“walking”, “sitting”, etc.) and low-level signals (raw sensor readings), can be developed manually to assist activity recognition. Although effective, this manual approach relies heavily on human domain expertise and is not scalable. In this paper, we address this limitation by proposing a machine learning method, SemNet, based on deep belief networks. SemNet automatically constructs semantic features representative of the axial bodily movements. Experimental results show that SemNet outperforms baseline approaches and is capable of learning features that highly correlate with manually defined semantic attributes. Furthermore, our experiments using a different model, namely deep convolutional LSTM, on household activities illustrate the broader applicability of semantic attribute interpretation to diverse deep neural network approaches. These empirical results not only demonstrate that such a deep learning technique is semantically meaningful and superior to its handcrafted counterpart, but also provides a better understanding of the deep learning methods that are used for Human Activity Recognition.

## 1. Introduction

Human activity recognition (HAR) through smartphones has been an indispensable component in mobile ubiquitous computing. As a foundation, HAR enables many context-aware applications and services (Chennuru et al., 2012; Wu et al., 2013; Wang et al., 2014). To recognize activities of a mobile user, various machine learning (ML) algorithms have been applied and engineered for specific application contexts (Bao and Intille, 2004; Huynh et al., 2008; Chen et al., 2021).

**Figure 1:**
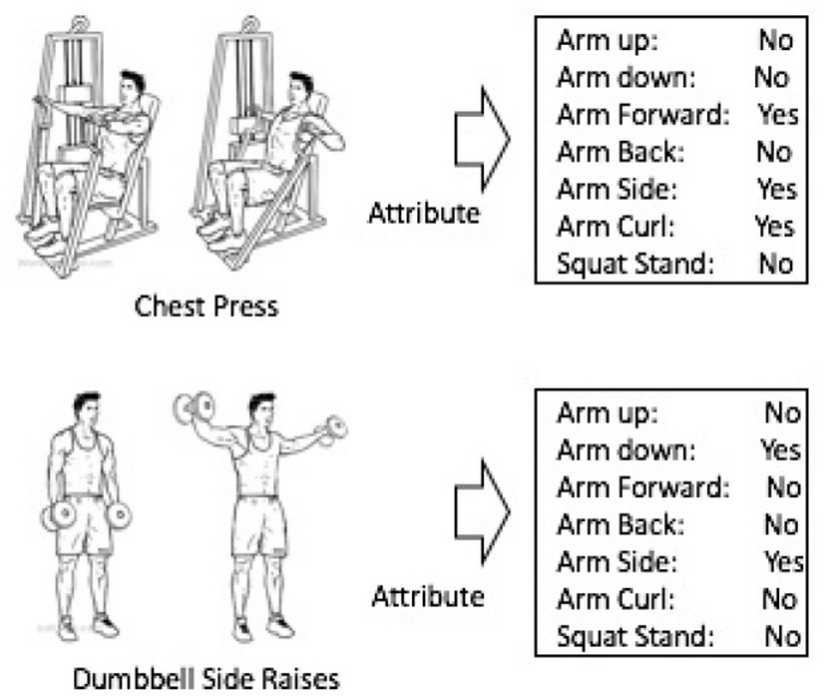
High-level activities can be represented by a set of semantic attributes.

Many existing ML methods use labeled training data for every single activity class that the HAR system aims to detect. However, this methodology omits some useful information. For example, rich structural information of the “chest press” activity as shown in Figure 1 can hardly be characterized by such a single class label. At the same time, most existing approaches have to enumerate all existing activity classes, and cannot recognize a previously unseen activity if there were no training samples for that activity (Cheng et al., 2013b). One popular solution to these challenges is to introduce semantic features that capture higher level concepts (Huynh et al., 2008; Cheng et al., 2013b). One approach to introduce such semantic features is by manually designing semantic attributes (Cheng et al., 2013a,b). This approach has also proven effective in computer vision (Farhadi et al., 2009; Liu et al., 2011; Mittelman et al., 2013). Researchers have also applied it in HAR and achieved satisfactory results (Cheng et al., 2013a,b).

Figure 1 illustrates the attribute concept in activity recognition. The workout activity “chest press” may be effectively represented by introducing a set of semantic attributes: “arm forward,” “arm side,” “arm curl,” and so forth.

The attribute representation can be obtained through the following steps: (1) an expert with domain knowledge defines a set of attributes, and each instance in the training dataset has to be labeled with the presence or absence of each attribute; (2) a classifier is trained for each of the attributes using the training data; (3) a feature selection scheme is applied on the attributes to create appropriate feature combination (Farhadi et al., 2009). However, obtaining these attributes is often time consuming and expensive since it requires much effort from test subjects, human annotators and domain experts. This demanding procedure also suffers from a scalability issue when new activities and new low-level features are present. Moreover, selecting attributes manually can be subjective and arbitrary, and may lead to non-discriminative features.

Some unsupervised learning algorithms attempt to construct semantic features instead of attributes. Some approaches rely on latent Dirichlet allocation (LDA) (Blei et al., 2003), which uses a set of topics to describe activities. LDA has been successfully applied in text analysis, information retrieval, computer vision (Lampert et al., 2009), and human activity recognition (Huynh et al., 2008). However, unlike words in text, activity signals have less clear semantic interpretations. Therefore, LDA has not been very successful in identifying semantic feature representations.

Another line of work is represented by deep neural networks, which learn a hierarchical set of features in an unsupervised or supervised manner. For example, the idea underlying Deep Belief Network (DBN) is to use restricted Boltzmann machine (RBM) (Hinton, 2002) as a building block. This enables the use of a greedy layer-wise learning procedure. RBM is a bi-partite undirected graphical model that is capable of learning a dictionary of patterns. These patterns are positively correlated with the observed input data. In computer vision, DBNs have achieved promising results (Mittelman et al., 2013). Further, many deep learning approaches have been applied in activity recognition task (Plötz et al., 2011; Zeng et al., 2014a, 2017, 2018; Chen et al., 2021). In this paper, we expand the RBM into a hierarchical representation, wherein relevant semantic concepts are revealed at the higher levels. Additionally, we use Indian buffet process (IBP) to train a sparse DBN, which helps to get more relevant semantic features and improve the results.

In order to identify the semantic concepts that are captured by the semantic features by a sparse DBN, we carry out experiments and evaluate the performance. By computing the correlation between learned features and each of the labeled attributes in the training set, we can evaluate the correspondences between the learned features and the labeled attributes. We demonstrate that we can find semantic concepts similar to attributes like “arm up” and “arm down,” even though no information with regards to these attributes was given during the training process. Improved accuracy further demonstrates that HAR applications can benefit from deep learning approaches.

We summarize our key contributions as follows:

- We propose an approach that uses a heterogeneous sparse DBN to extract semantic feature representation without using any domain knowledge.
- We also demonstrate that learned features carry appropriate semantic meaning by calculating and evaluating correlation with available manually defined attributes.
- We demonstrate semantic correlation of attributes for two different models on two datasets: (1) the proposed sparse DBN on Exercise Activity dataset (Cheng et al., 2013b), and (2) a deep convolutional LSTM on Opportunity Human Activity Recognition dataset (Chavarriaga et al., 2013).

The paper is organized as follows. We begin with a survey of related work and discuss how it compares to our work. Next, we present our approach built on the restricted Boltzmann machine and Deep Belief Networks (DBNs). Furthermore, we propose a sparse DBN-based mechanism that enhances the results. We thereafter present experimental results and analysis. Finally, we conclude and discuss future research directions.

## 2. Related work

In the field of mobile, wearable, and pervasive computing, extensive research has been conducted to recognize human activities (Bao and Intille, 2004; Blanke and Schiele, 2010; Peng et al., 2011; Plötz et al., 2011; Cheng et al., 2013a,b; Zeng et al., 2014a,b, 2017, 2018; Yu et al., 2016; Pan et al., 2017). One line of research in this field starts with Bao and Intille (2004), who placed accelerometers on different body positions to recognize daily activities such as “walking,” “sitting,” and “watching TV.” Since then, researchers have been devoted to improving recognition accuracy. Many of them investigated underlying structural representations of activities. For example, Peng et al. (2011) apply the hidden Markov model (HMM) to model activities using one latent layer.

The idea of latent structure was extended for recognizing previously unseen activities. Cheng et al. (2013b,a) leverage zero-shot learning (Palatucci et al., 2009) in the NuActiv approach, using predefined semantic attributes to predict new activities. Essentially, the manually defined attributes can be regarded as semantic features. The introduction of such features have been proven effective in computer vision, for instance in object recognition (Lampert et al., 2009; Russakovsky and Fei-Fei, 2010; Liu et al., 2011).

Manually defining attributes, however, is time-consuming and expensive. To address these drawbacks, Mittelman et al. (2013) propose the Beta-Bernoulli process restricted Boltzmann machine (BBP-RBM) to learn semantic features for object recognition. In HAR, there are similar approaches attempting to construct semantic features using latent Dirichlet allocation (LDA) (Huynh et al., 2008). Huynh et al. showed that LDA-based approaches, however, are limited to features that have high correlation with the activities to be recognized (Huynh et al., 2008). Deep neural networks represent another line of study to learn hierarchical features in an unsupervised manner. Plötz et al. (2011) applied the RBM to extract features from accelerometer data. Zeng et al. (2014a) took advantage of convolutional neural network to preserve local dependency and scale invariant features to achieve better recognition performance. In contrast, we are, in this paper, able to leverage a DBN to learn relevant semantic features pertaining to HAR without requiring manually defined attributes.

To avoid overfitting in training, sparsity is introduced into deep neural networks (Lee et al., 2007; Glorot et al., 2011; Salakhutdinov et al., 2013; Srivastava et al., 2014). Advantages of sparsity also include information disentangling and efficient variable-size representation (Glorot et al., 2011). One popular sparsity technique is dropout (Srivastava et al., 2014), which randomly removes some nodes in each iteration during the training procedure. Lee et al. (2007) set thresholds in the node selection phase of RBM to enforce sparsity penalty. Mittelman et al. (2013) use a Beta-Bernoulli process over the RBM to remove some nodes. Bhattacharya et al. (2014) use a sparse-coding framework to build a feature space codebook onto which the transportation activities in their experiment were mapped. In this work, we also introduce heterogeneous sparsity into our DBN in order to achieve superior results.

Deep neural networks, implementing various types of CNNs, LSTMs, etc. have achieved state-of-the-art results on HAR recently (Nweke et al., 2018; Chen et al., 2021; Erdaş and Güney, 2021). Current works typically focus on multi-modal sensing, i.e., performing activity recognition using multiple different sensors such as accelerometers, gyroscopes, etc. EmbraceNet (Choi and Lee, 2019) uses separate docking and embracement layers to effectively perform sensor fusion. Many works successfully combine CNNs and RNNs to perform complex activity recognition (Ordóñez and Roggen, 2016; Zhao et al., 2018; Xu et al., 2019). The authors in Hassan et al. (2018) perform smartphone-based activity recognition using a DBN and SVM-based model. Apart from a plethora of supervised learning approaches along these lines, a few works also leverage unsupervised learning and deep generative models for HAR. Some of them use different variants of autoencoders, like stacked autoencoders (Chikhaoui and Gouineau, 2017), stacked denoising autoencoders (Gu et al., 2018) and CNN autoencoders (Zeng et al., 2017). A recent work has proposed using deep variational autoencoders (VAEs) (Bai et al., 2019) to learn highly effective representations of activity time sequences using unlabeled data.

## 3. DBN with heterogeneous sparsity for learning semantic features

Our proposed deep learning methodology for human activity recognition is based on deep belief networks (DBNs) and we use the outputs of the last hidden layer to assess correlations with manually defined mid-level features. The main reason to use DBNs here is to introduce prior to the HAR methodology that eventually enables the model to better capture the mid-level semantic features. In this section, we describe the key components of our proposed DBN model, including Restricted Boltzmann Machine (RBM), different types of sparsity such as dropout and Indian Buffet Process (IBP) and the training procedure.

### 3.1. Standard restricted boltzmann machine

An RBM is a two-layer undirected probabilistic graph, in which the visible input layer contains a set of binary or real valued units {*v*_1_, …, *v*_*N*_*v*__} and the hidden layer is composed of a set of binary units {*h*_1_, …, *h*_*N*_*h*__}. Here, *N*_*v*_ and *N*_*h*_ are the numbers of visible units and hidden units, respectively. Connections are only allowed between the visible layer and the hidden layer. Let $v={\left[v_{1},\ldots ,v_{N_{v}}\right]}^{T}$ and $h={\left[h_{1},...,h_{N_{h}}\right]}^{T}$, where *T* denotes the transpose. The energy function of RBM is defined as


(1) $$\begin{array}{l} E\left(v,h\right)=-h^{T}Wv-b^{T}v-c^{T}h \end{array}$$

where *W* = [*w*_*ji*_]*N*_*h*_×*N*_*v*_ is the weight matrix, *b* = [*b*_*i*_]*N*_*v*×1_ is the bias of visible units and *c* = [*c*_*j*_]*N*_*h*_×1 is the bias of hidden units. Then the joint probability distribution of *v* and *h* with σ as the activation function is


(2) $$\begin{array}{l} p\left(h_{k}|v\right)=\sigma \left(w_{k,i}v_{i}+b_{k}\right) \end{array}$$

(3) $$\begin{array}{l} p\left(v_{i}|h\right)=\sigma \left(w_{k,i}h_{k},+c_{i}\right) \end{array}$$

The log likelihood function corresponding to the visible units is given by


(4) $$\begin{array}{l} P\left(v\right)=\frac{1}{Z}\underset{h}{\sum }\left(-E\left(v,h\right)\right) \end{array}$$

where *Z* is the normalization factor.

We denote the parameters of RBM by θ = {*W, b, c*}. The derivative of the log-likelihood of visible units [P(*v*)] with respect to model parameter θ can be written as


(5) $$\begin{array}{l} \frac{\partial P\left(v\right)}{\partial \theta }={\text{E}}_{data}\left(-\frac{\partial E\left(v,h\right)}{\partial \theta }\right)-{\text{E}}_{model}\left(-\frac{\partial E\left(v,h\right)}{\partial \theta }\right) \end{array}$$

where *E*_*data*_(·) and *E*_*model*_(·) denote the expectations of the data distribution and the model distribution, respectively. Computing the function $\frac{\partial P\left(v\right)}{\partial \theta }$ in (5) exactly is intractable because the closed form of the model distribution remains unknown. However, the derivative can be approximately computed by Contrastive Divergence (CD) (Hinton, 2002). With CD, the locally optimal solutions of model parameters θ can be attained by gradient descents.

### 3.2. RBM with random dropout sparsity

Dropout training controls overfitting by randomly omitting subsets of features at each iteration of a training procedure (Hinton et al., 2012). Formally, we can use *F* = *f*_1_, …, *f*_*K*_, to represent an indicator vector, *F* ∈ 0, 1. Each *f*_*k*_ is generated according to a uniform distribution, *f*_*k*_ ~ *U*(γ). In each iteration, *F* is enforced on each input layer to remove nodes using *f*_*k*_.

### 3.3. Indian buffet process

The Indian buffet process (IBP) can be applied to generate a binary indicator vector with similar 0/1 patterns. It is natural to combine with the RBM probability model. We use **z**_*IBP*_ = [*z*_1_, …, *z*_*K*_] to denote the indicator vector. We assume the two-parameter IBP (Ghahramani et al., 2007), and use *Z* ~ *IBP*(α, β)to indicate the vector $Z_{IBP}\in {\left\{0,1\right\}}^{K}$. Specifically, the indicator vector *Z* is generated according to a Beta-Bernoulli process as follows:


(6) $$\begin{array}{l} \begin{array}{l} \pi \sim Beta\left(\alpha /K,\beta \left(K-1\right)/K\right),\\ z_{k}\sim Bernoulli\left(\pi_{k}\right) \end{array} \end{array}$$

Where α, β are positive parameters, and we use the notation $\pi ={\left[\pi_{1},\ldots \pi_{K}\right]}^{T}$ for the parameters of the Bernoulli distribution. It is implied from (6) that if π_*k*_ is close to 1 then *z*_*k*_ is more likely to be 1, and vice versa. The form of the parameters of Beta distribution implies that for a sufficiently large *K* and a reasonable choice of α and β, most π_*k*_ will be close to zero, which implies a sparsity constraint on *z*_*k*_.

### 3.4. RBM with IBP sparsity

In this section, we enhance the generalization ability of RBM from a different perspective - by enforcing constraints on the nodes of hidden layer. Dropout increases sparsity by removing hidden nodes uniformly in each training epoch. However, by leveraging IBP, we demonstrate we are able to obtain better sparse features due to IBP's grouping characteristic (Banos et al., 2014).

The binary selection vector $z={\left[z_{1},\ldots ,z_{K}\right]}^{T}$ is used to choose which of the *K* hidden units should be allowed to remain activated. Our approach is to define an undirected graphical model in the form of a factor graphical model. Using the binary selection vector mentioned above, we have a new energy function


(7) $$\begin{array}{l} E\left(v,h,z\right)=-{\left(z⊗h\right)}^{T}Wv-b^{T}\left(z⊗h\right)-c^{T}v, \end{array}$$

where ⊗ denotes element-wise vector multiplication. With the new energy function, we can define


(8) $$\begin{array}{l} g_{1}\left(v,h,z\right)=e^{-E\left(v,h,z\right)} \end{array}$$

Since the binary selection vector is created *via* Beta-Bernoulli Process, its distribution function can be described as


(9) $$\begin{array}{l} \begin{array}{c} g_{2}\left({\left\{z^{j}\right\}}_{j=1}^{M},\pi \right)=\overset{K}{\underset{k=1}{\prod }}\pi_{k}^{\overset{M}{\underset{j=1}{\sum }}z_{k}^{j}}{\left(1-\pi_{k}\right)}^{\overset{M}{\underset{j=1}{\sum }}\left(1-z_{k}^{j}\right)}\\ \;\times \pi_{k}^{\alpha /K-1}{\left(1-\pi_{k}\right)}^{\beta \left(K-1\right)/K-1} \end{array} \end{array}$$

where *j* denotes the index of the training sample, and *M* represents the number of training samples.

Using the training factor graph, the PDF for IBP-RBM is


(10) $$\begin{array}{l} \begin{array}{l} p\left({\left\{v^{j},h^{j},z^{j}\right\}}_{j=1}^{M},\pi \right)\propto \overset{M}{\underset{j=1}{\prod }}g_{1}\left(v^{j},h^{j},z^{j}\right)\\ {\;g}_{2}\left({\left\{z^{j}\right\}}_{j=1}^{M},\pi \right) \end{array} \end{array}$$

### 3.5. IBP-RBM inference

Inference in IBP-RBM can be estimated by Gibbs sampling. The joint posterior PDF of *h* and *z* can be sampled as below


(11) $$\begin{array}{l} p(h_{k}=a,z_{k}=b|v_{k},\pi_{k})\propto \{\begin{array}{ll} \pi_{k}e^{\sum_{i}w_{k,i}v_{i}} & a=1,b=1\\ \pi_{k} & a=0,b=1\\ 1-\pi_{k} & a=0,b=0\\ 1-\pi_{k} & a=1,b=0 \end{array} \end{array}$$

Then the posterior PDF of π takes the form


(12) $$\begin{array}{l} \begin{array}{l} \pi_{k}\sim \\ \;Beta\left(\alpha /K+\overset{M}{\underset{j=1}{\sum }}z_{k},\beta \left(K-1\right)/K+\overset{M}{\underset{j=1}{\sum }}\left(1-z_{k}\right)\right) \end{array} \end{array}$$

Sampling from the posterior PDF of the visible layer is performed in a similar manner as described in standard RBM.

### 3.6. DBN with heterogeneous sparsity

**Figure 2:**
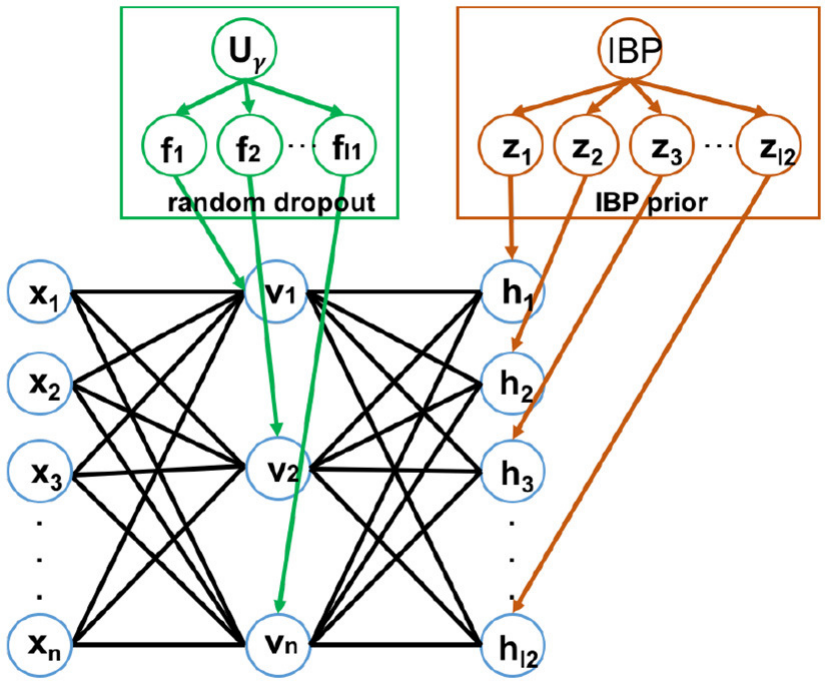
The structure of Heterogeneous Sparse DBN.

Once a layer of the network is trained, the parameters *w*_*ij*_, *b*_*j*_, *c*_*i*_'s are frozen and the hidden unit values are inferred from the given data. These inferred values act as the “data” that will be used to train the next higher layer in the network. We use dropout on the first hidden layer and use IBP on the second hidden layer, which injects heterogeneous sparsity to the DBN (HSparseDBN). Figure 2 shows the structure of the HSparseDBN model. The details of our procedure are summarized in Algorithm 1.

**Algorithm 1:**
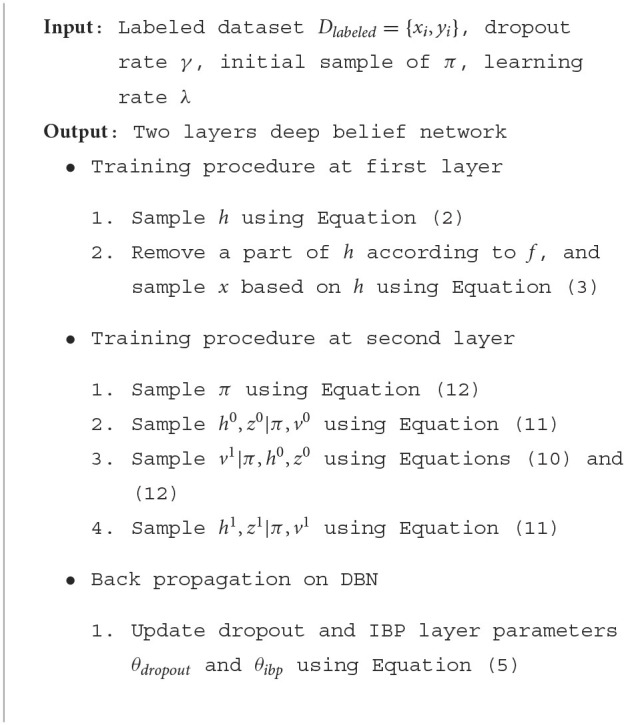
Heterogeneous sparse DBN (HSparseDBN) training procedure.

## 4. Experimental results and analysis

In this section, we present our experiment setup and evaluate performance of our approach.

### 4.1. Experimental procedure

We mainly use the Exercise Activity dataset for our evaluation. The dataset contains human activities in different contexts and have been recorded using tri-axial accelerometers. The sensor data is segmented using a sliding window with a size of 64 continuous samples and 50% overlap. We experiment with all our deep learning algorithms on a computer equipped with a Tesla K20c GPU and 64G memory. Other computations run on the same computer but on an Intel Xeon E5 CPU. Throughout this section, we use two hidden layer DBN, with 2,048 and 1,024 nodes in the first and second hidden layer, respectively. The dropout rate in the first hidden layer is 0.3 and the parameter values for IBP in the second layer are α = 1, β = 5. The other parameters *W, b, c* in the network were initialized by drawing from a zero mean Gaussian with standard deviation 0.005. We also use weight decay and momentum in our networks. The regularization parameters are 0.998 and 0.95. We use rectified linear unit (ReLU) as the activation function.

Additionally, we also train a Deep Convolutional LSTM model on Opportunity Human Activity Recognition dataset to increase diversity in the model and dataset. In contrast to Exercise Activity dataset that involves exercises, Opportunity dataset involves regular day-to-day activities such as opening/closing a door. This experiment is described further in the last subsection on exploring the generality of semantic interpretation beyond DBNs.

#### 4.1.1. Exercise activity dataset

**Figure 3:**
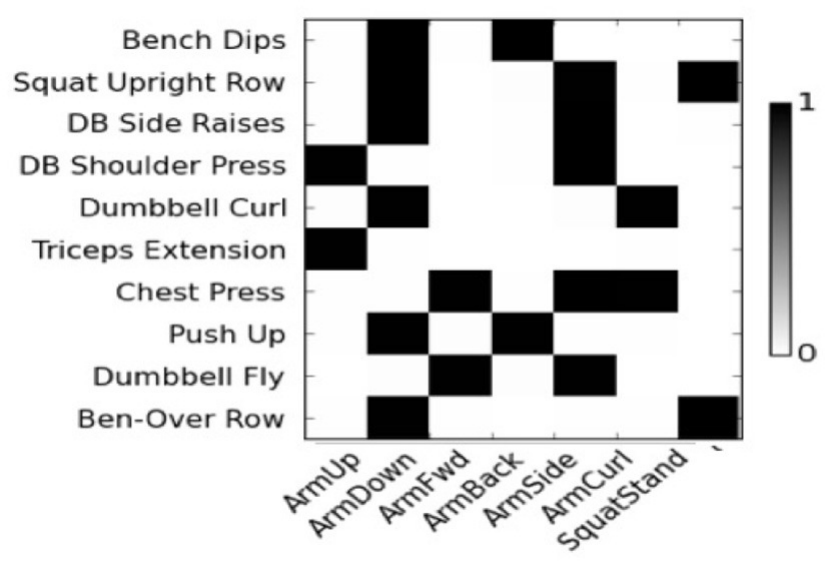
Correlation between human activities (in rows) and attributes (in columns) for the Exercise Activity dataset.

In the Exercise Activity dataset (Cheng et al., 2013b), 20 test subjects were asked to perform a set of 10 exercise activities (Chang et al., 2007). Each subject was equipped with three sensor-enabled devices: a Nexus S 4G phone in an armband, a MotoACTV wristwatch, and a second MotoACTV clipped to the hip. The dataset contains accelerometer and gyroscope data collected at 30 Hz sampling rate. For feature extraction, the sliding window size is empirically set to 1 s with 50% overlap based on a leave-one-out cross-validation test. The dataset contains around 8,000 instances. Figure 3 plots the correlation values between human activities and semantic attributes, as derived from the Exercise Activity dataset. It depicts how exercise activities are strongly associated with certain arm/leg movements, thus demonstrating the potential for semantic feature extraction.

### 4.2. Recognition accuracy

In Table 1, we compare the accuracy values obtained for Exercise Activity dataset when using features learned from the training set using dropout DBN, Heterogeneous Sparse DBN (HSparseDBN), statistical features, and combinations of DBN with statistical features. The statistical features are obtained by calculating mean across the input using a sliding window. The classifier used here is a multi-class linearSVM (Fan et al., 2008). When performing leave-one-out validation, only one user is used as test data and the rest form training data.

**Table 1:** Accuracy comparison between several methods, all using linearSVM classifier for the Exercise Activity dataset.

**Features**	**Accuracy (%)**
Statistical	77.30
DropoutDBN	81.56
HSparseDBN	82.30
Statistical+DropoutDBN	84.38
Statistical+HSparseDBN	**85.72**

Bold value represents the highest valued result in the table.

From Table 1, we see that accuracy is higher when using DBN features compared to using just statistical features. This suggests that DBN is able to capture more useful and relevant semantic features. Furthermore, the accuracy of DBN + statistical features is higher than when using only DBN features or statistical features. This implies that DBN alone does not capture all of the features and that the statistical features are complementary to the DBN features. We also note that using HSparseDBN improves accuracy over DropoutDBN, thus demonstrating superior generalization ability of sparse features due to IBP's grouping characteristic. We conclude that the HSparseDBN + statistical features method benefits from both heterogenous sparsity of DBN and statistical features and hence outperforms all other methods.

### 4.3. Learned features vs. semantic attributes

In this section, we evaluate the degree to which the features learned using the DBN can capture semantic concepts. For each feature and label attribute pair, we compute the correlation and find the most correlated DBN-based feature for each semantic attribute.


(13) $$\begin{array}{l} r_{attri,dbn}=\frac{\sum_{i=1}^{M}\left(x_{attri}-{\bar{x}}_{attri}\right)(x_{dbn}-{\bar{x}}_{dbn})}{\sqrt{\sum_{i=1}^{M}{\left(x_{attri}-{\bar{x}}_{attri}\right)}^{2}\sum_{i=1}^{M}{\left(x_{dbn}-{\bar{x}}_{dbn}\right)}^{2}}} \end{array}$$

**Figure 4:**
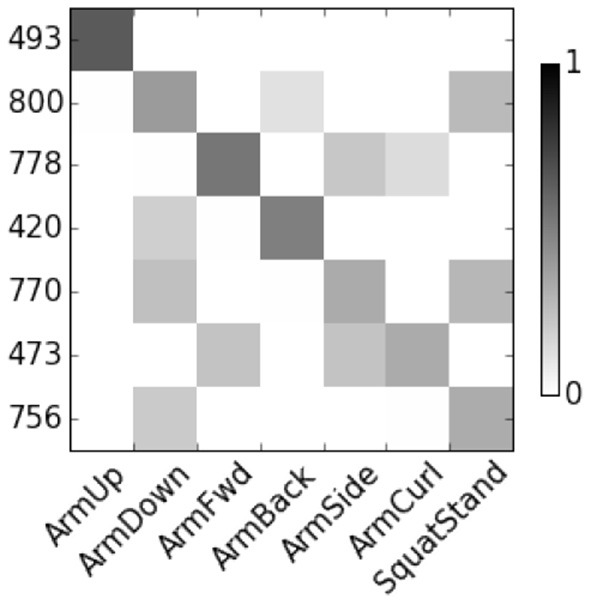
Correlation between DropoutDBN features and attributes for User 8.

**Figure 5:**
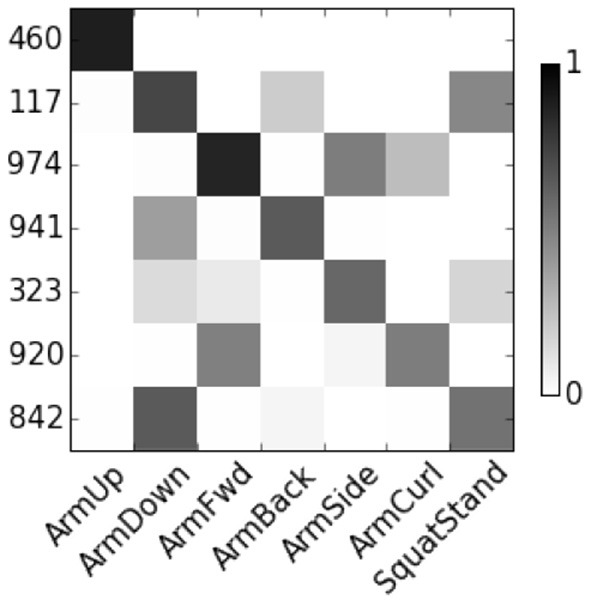
Correlation between HsparseDBN features and attributes for user 8.

Figure 4 show the correlation score of User 8. The features are represented by node numbers on the left. Most of dropout DBN features have a score greater than 0.5. The correlation between HSparseDBN features and attributes is shown in Figure 5. The result shows that all the corresponding features have high correlation scores. This supports our hypothesis that the learned features from DBN can capture important relevant semantic concepts, and demonstrates the benefit of using heterogeneous sparsity.

### 4.4. Domain adaption

**Figure 6:**
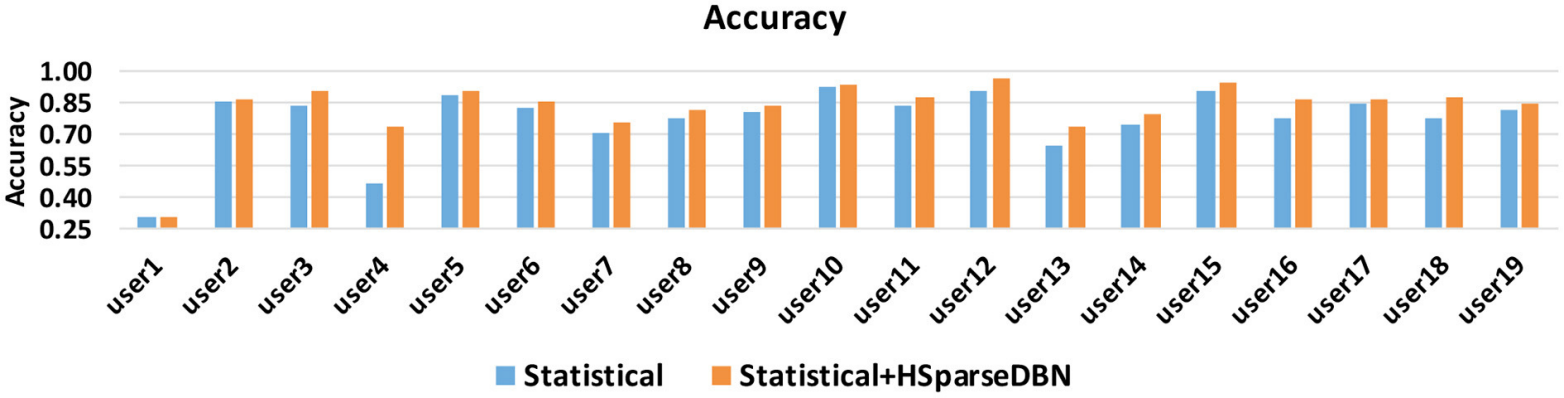
Accuracy of statistical features and statistical + HSparseDBN features, for users in Exercise Activity dataset.

An important aspect of evaluating the features is the degree to which they generalize well across different users, even if their distributions are different from each other. In this case the distribution of training set and test set is no longer i.i.d. In this subsection, we look into the accuracy of test user in the leave-one-out validation. In the test procedure, a test set contains certain instances from only one user, and the rest of users combine to form the training set. Since we already observed that Statistical+HSparseDBN performs best on average on these datasets, we compare the accuracy when using statistical features and the combination of statistical and HSparseDBN features. The results over 19 individual users are shown in Figure 6. From the results, we can see that statistical+HSparseDBN consistently outperforms statistical features alone, except for user 1 (31.07 vs. 30.72%).

### 4.5. Exploring the generality of semantic interpretation

In addition to the previous experiments discussed above, we use another dataset to explore the applicability of semantic interpretation more generally in other types of deep neural network models. In order to do so, we utilize Opportunity Challenge dataset and a deep convolutional LSTM model, both of which are described below.

#### 4.5.1. Opportunity challenge dataset

This dataset contains regular day-to-day human activities performed within a home environment with readings performed by motion sensors located on the body, different objects in the environment as well as ambient sensors. Recordings are taken from four different subjects over multiple runs with scripted as well as unscripted sequence of activities such as opening/closing the door, opening/closing the refrigerator, preparing coffee, toggling the light switch, etc. These are provided as 242 sensor attributes and classified as 17 different “mid-level gesture” activities in the dataset. Note that this definition is different from the “mid-level attributes” as used in this paper. The dataset also provides annotations for 13 “low-level” activities (for each arm) like unlock, lock, reach, sip, etc. which together combined with the objects provide the mid-level activities. For the purpose of our experiment, we term these 13 low-level activities as the semantic mid-level features to be learnt by a model trying to classify the dataset's 17 mid-level gesture activities. Also, it is to be noted that we only use a subset of the attributes as done in the Opportunity Challenge, which selects 113 out of the total 242 attributes.

#### 4.5.2. Deep convolutional LSTM

We use a similar deep convolutional LSTM model as in Ordóñez and Roggen (2016) with four convolutional layers followed by two LSTM layers and a final fully connected layer, implemented in PyTorch. The last hidden layer, i.e., the second LSTM layer, consists of 128 nodes and implements dropout sparsity with 0.5 probability. The data to the input layer is provided using a sliding window of 24 with an overlap of 12, with 113 input channels. The convolutional layers all have 64 output channels and use 5x5 kernels. The final fully connected layer is used to classify 18 classes of dataset's mid-level activities. The additional one class is used to classify unidentified or ambiguous activities.

#### 4.5.3. Semantic correlation

**Figure 7:**
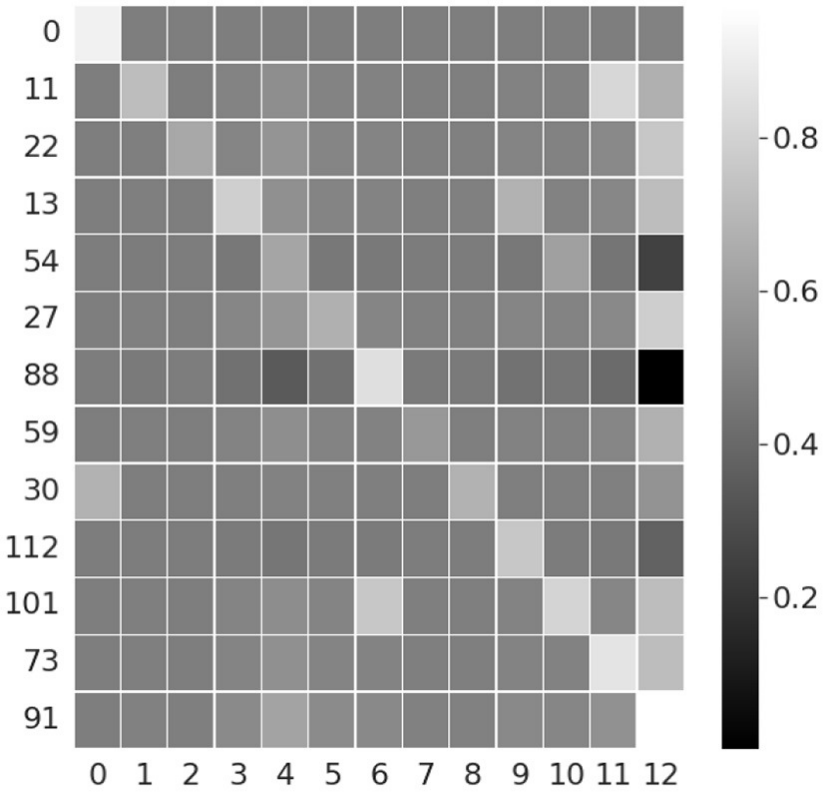
Correlation between DeepConvLSTM features and 13 semantic attributes from Opportunity dataset. Y axis plots the hidden dimensions correlated with the semantic attributes on X axis.

We train the above model for 10 epochs with SGD optimizer and cross-entropy loss, and achieve matching results as original implementation, with 84.35% validation accuracy. Once trained, we take the 128-length feature vector from the second LSTM hidden layer (the last hidden layer in the network), and calculate correlation across the 13 semantic mid-level features. The hidden dimensions with the highest correlations are plotted in Figure 7. It can be seen from the figure that certain hidden layer nodes have high correlation with the corresponding semantic features (can be seen along the diagonal in Figure 7), thus implying the capability of the network to capture semantic interpretation. As this experiment is performed on a deep convolutional LSTM and Opportunity dataset with different type of activities compared to our earlier experiment using the proposed DBN and Exercise Activity dataset, it demonstrates the generality of the semantic capabilities of deep neural networks.

## 5. Conclusion and future work

In this paper, we demonstrate that deep neural networks can capture semantic concepts. We introduce a new method, called SemNet, for learning semantic feature representation using dropout and Indian buffet process DBN, which can avoid overfitting and group similar features. We use Exercise Activity dataset for our experiments and are able to achieve promising results. We also study the semantic concepts by calculating the correlation between manually defined attributes and learned features, using which we show that many of the extracted features have semantic meanings. Additionally, we also demonstrate semantic correlation on a completely different type of deep model, convolutional LSTM, on a different dataset consisting of regular daily household activities. As future work, we will test the proposed method on more datasets and examine the inference process of semantic topics. We also intend on exploring the efficacy of Recurrent Neural Networks and their sequence modeling capability for learning features with semantic meanings. We further intend to leverage variational autoencoders (VAEs) and leverage the learned encoder output to extract semantic correlation with attributes.

## Data availability statement

The original contributions presented in the study are included in the article/supplementary material, further inquiries can be directed to the corresponding author/s.

## Author contributions

SV, HN, and MZ: study conception and design. MZ: data collection. SV, HN, MZ, and CT: analysis and interpretation of results. HN, MZ, SV, OM, and JS: draft manuscript preparation. OM and JS: critical revision of the article. All authors reviewed the results and approved the final version of the manuscript.

## Conflict of interest

The authors declare that the research was conducted in the absence of any commercial or financial relationships that could be construed as a potential conflict of interest.

## Publisher's note

All claims expressed in this article are solely those of the authors and do not necessarily represent those of their affiliated organizations, or those of the publisher, the editors and the reviewers. Any product that may be evaluated in this article, or claim that may be made by its manufacturer, is not guaranteed or endorsed by the publisher.
