# Corrigendum: SemNet: Learning semantic attributes for human activity recognition with deep belief networks

**DOI:** 10.3389/fdata.2023.1170820

**Published:** 2023-03-10

**Authors:** Shanmuga Venkatachalam, Harideep Nair, Ming Zeng, Cathy Shunwen Tan, Ole J. Mengshoel, John Paul Shen

**Affiliations:** ^1^Department of ECE, Carnegie Mellon University, Pittsburgh, PA, United States; ^2^Department of ECE, Anderson School of Management, University of California, Los Angeles, Los Angeles, CA, United States; ^3^Department of Computer Science, Norwegian University of Science and Technology (NTNU), Trondheim, Norway

**Keywords:** human activity recognition, deep belief networks, semantic mid-level features, ubiquitous computing, multimodal sensing, artificial intelligence, internet of things

## Incorrect Affiliation

In the published article, there was an error regarding the affiliation for Ole J. Mengshoel. Instead of being affiliated with “Department of ECE, Carnegie Mellon University, Pittsburgh, PA, United States”, they should be affiliated with “Department of Computer Science, Norwegian University of Science and Technology (NTNU), Trondheim, Norway.”

## Incorrect Correspondence

In the published article, there was an error in the correspondence. The correct corresponding author is “Ole J. Mengshoel” instead of “Harideep Nair.”

The authors apologize for these errors and state that this does not change the scientific conclusions of the article in any way. The original article has been updated.

